# Fat-mass and obesity-associated gene polymorphisms and weight gain after risperidone treatment in first episode schizophrenia

**DOI:** 10.1186/1744-9081-10-35

**Published:** 2014-10-02

**Authors:** Xueqin Song, Lijuan Pang, Yufang Feng, Xiaoduo Fan, Xue Li, Wei Zhang, Jinsong Gao, Jianjiang Zhang, Katlyn Nemani, Hua Zhang, Luxian Lv

**Affiliations:** The first Affiliated Hospital/Zhengzhou University, Zhengzhou, China; Qingdao Mental Health Center, Qingdao, China; Psychotic Disorders Program, UMass Memorial Medical Center/University of Massachusetts Medical School, Worcester, MA UK; Tufts University School of Medicine, Boston, MA UK; Henan Province Biological Psychiatry Key Laboratory, Xinxiang Medical University, Xinxiang, China; Henan Province Mental Hospital, the Second Affiliated Hospital/Xinxiang Medical University, Xinxiang, China

**Keywords:** FTO gene, Weight gain, Schizophrenia, Single nucleotide polymorphism (SNP)

## Abstract

**Background:**

Obesity induced by antipsychotics severely increases the risk of many diseases and significantly reduces quality of life. Genome Wide Association Studies has identified fat-mass and obesity-associated (FTO) gene associated with obesity. The relationship between the FTO gene and drug-induced obesity is unclear.

**Method:**

Two hundred and fifty drug naïve, Chinese Han patients with first-episode schizophrenia were enrolled in the study, and genotyped for four single nucleotide polymorphisms (SNPs rs9939609, rs8050136, rs1421085 and rs9930506) by the polymerase chain reaction-restriction fragment length polymorphism (PCR-RFLP) and direct sequencing. Body weight and body mass index (BMI) were measured at baseline and six months after risperidone treatment.

**Results:**

At baseline, body weight and BMI of TT homozygotes were lower than those of A allele carriers in rs9939609; body weight of AA homozygotes was higher than those of G allele carriers in rs9930506 (p’s < 0.05). After 6 months of risperidone treatment, body weight and BMI of TT homozygotes were lower than those of A allele carriers in rs9939609 (p’s <0.01); body weight and BMI of CC homozygotes were lower than those of A allele carriers in rs8050136 (p’s < 0.05); body weight of AA homozygotes was higher than those of G allele carriers in rs9930506 (p’s < 0.05). After controlling for age, gender, age of illness onset, disease duration, weight at baseline and education, weight gain of TT homozygotes at 6 months remained to be lower than those of A allele carriers in rs9939609 (p < 0.01); weight gain of CC homozygotes at 6 months was lower than those of A allele carriers in rs8050136 (p = 0.01). Stepwise multiple regression analysis suggested that, among 4 SNPs, rs9939609 was the strongest predictor of weight gain after 6 months of risperidone treatment (p = 0.001).

**Conclusions:**

The FTO gene polymorphisms, especially rs9939609, seem to be related to weight gain after risperidone treatment in Chinese Han patients with first episode schizophrenia.

## Background

Schizophrenia is a psychiatric disorder that is associated with impairment in social and occupational functioning [1]. The use of antipsychotics, especially atypical antipsychotics (AAPs), is associated with serious adverse metabolic effects, including weight gain, hyperlipidemia and glucose intolerance [2], and other [3]. But researches also suggested that patients taking AAPs do not necessarily develop these adverse effects, suggesting that differential genetic vulnerability may play a role in antipsychotic-associated metabolic side effects.

The advent of Genome Wide Association Studies (GWAS) emerged as a powerful approach to identify genetic variants associated with common diseases [4]. GWAS has identified a group of loci associated with obesity [5]. In 2007, Frayling et al. [6] and Scuteri et al.[7] found a strong association between common single nucleotide polymorphisms (SNPs) in the first intron of the fat-mass and obesity-associated (FTO) gene on the chromosome 16q12.2, and the risk of obesity. Subsequently, many studies have unequivocally replicated the relationship between FTO gene variants and obesity in children and adults across different populations [8–15], including Han Chinese [13, 16–18].

The risk of weight gain is variable among different antipsychotics. Olanzapine and clozapine are associated with the highest risk of weight gain, followed by chloropromzaine, resperidone, and quetiapine [19]. Ziprasidone [20] and aripiprazole belong to the group with the lowest risk of weight gain. The rank order of antipsychotics for inducing weight gain is consistent with that of their metabolic side effects [21], supporting the idea that drug’s risk for adverse metabolic changes are strongly associated with its potency for increasing obesity. Risperidone is a common drug used for schizophrenia treatment in China. As we known, Variants of FTO gene (such as rs9939609, rs8050136, rs1421085 and rs9930506) have been consistently reported to be associated with obesity in recent studies [7, 12, 22, 23], but there have been no studies tested the potential role in antipsychotic-related obesity except rs9939609.

The aim of this study was to examine the relationship between FTO gene polymorphisms (rs9939609, rs8050136, rs1421085 and rs9930506) and weight gain after 6 months of risperidone monotherapy in drug-naïve, Chinese Han patients with schizophrenia.

## Methods

### Subjects

All subjects provided written informed consent to participate in the study, which was approved by the Ethics Committee of the First Affiliated Hospital of Zhengzhou University and Henan Mental Hospital. Chinese Han inpatients between 18 and 50 years old diagnosed with first-episode schizophrenia (disease duration less than 2 years) were recruited. Patients were diagnosed with first-episode schizophrenia according to the criteria of Diagnostic and Statistical Manual of Mental Disorders, Fourth Edition (DSM-IV), and were never previously treated with antipsychotic medications or other psychotropics. The diagnosis of schizophrenia was further determined by two research psychiatrists (X.S. in the First Affiliated Hospital of Zhengzhou University and L.L. in Henan Mental Hospital) using the Structured Clinical Interview for DSM-IV Axis I Disorders. Exclusion criteria included ongoing infections or allergies, history of alcohol or other substance use, pregnancy, known medical conditions that might affect metabolism, history of diabetes or lipid disorder, use of anti-diabetic or lipid-lowering agents or special diets to lower glucose or lipid levels. A complete medical history was obtained from all subjects. All subjects were treated in the two hospitals, and underwent daily physical examination and weekly routine laboratory tests.

After baseline assessment, all patients were treated with risperidone with the dose ranging from 2 mg to 6 mg per day based on the clinical judgment of treating psychiatrists. No other medication was allowed during the study except benzodiazepines for insomnia and anticholinergic agents for dystonia reaction.

### Clinical assessment

Anthropometric assessment was performed at baseline and 6 months after risperidone treatment. Anthropometric measures for each participant were taken after an overnight fast while the subject wore light clothing and no shoes. Weight (kg) and height (m) were measured, and body mass index (BMI) was calculated for all subjects. Symptoms of schizophrenia were assessed using the Positive and Negative Syndrome Scale (PANSS). The clinical assessment was administered by two clinical psychiatrists (L.P. in the First Affiliated Hospital of Zhengzhou University and X.L. in Henan Mental Hospital) who had attended a training session for the proper use of PANSS to ensure the consistency and reliability of the ratings throughout the study. A correlation coefficient above .8 was maintained for the PANSS total score after repeated assessments.

### Genotyping

DNA samples were isolated from peripheral blood using the QIAamp DNA blood kit (QIAGEN GmbH, Hilden, Germany). Genotyping of the FTO SNPs (rs9939609, rs8050136 and rs1421085) was performed using direct sequencing. The PCR-RFLP assay was used to determine the genotype of SNP rs9939506. PCR was carried out in a DNA thermal cycler (Biometra, Goettingen, Germany) using the primers: forward: 5'-CAAAGGTGGGCATAGAGATTG-3'; reverse: 5'-AAGGATTTCTGAGGGACACA-3'. PCR was performed after the first denaturation at 95°C for 2 min; each cycle consisted of denaturation at 95°C for 20 sec, annealing at 62°C for 20 sec, and extension at 72°C for 30 sec. The number of total PCR cycles was 30. To assess genotyping reproducibility, randomly selected 20% DNA samples were re-genotyped with 100% concordance.

### Statistical analysis

The Hardy-Weinberg equilibrium (HWE) and linkage disequilibrium (LD) test of SNPs were done by using the PLINK program [24] and SHEsis. The data were analyzed using SPSS 20.0 (SPSS Inc., Chicago, IL). Demographics and clinical measures were reported using descriptive statistics. Comparisons between genotypes were performed using the Student’s t-test for continuous variables and *χ*^2^ test for categorical variables. Analysis of covariance was used to examine weight gain at 6 months of risperidone treatment after controlling for potential confounding variables. Further, stepwise multiple regression model was used to examine the relative predictive value of gene loci for the risk of weight gain. A p value of less than 0.05 (2-tailed) was used for statistical significance.

## Results

Two hundred and fifty first episode, drug naïve schizophrenia patients were enrolled in the study, and 237(94.8%) completed the follow-up assessment after 6 months of risperione treatment (Table 1). Thirteen patients withdrew from the study for various reasons before they reached the 6-month time point (relocation, 5; transportation difficulty, 6; side effects, 2).

**Table 1:** **Demographic and clinical characteristics of the study sample**

Variables	N or Mean ± SD
Male/female (n)	128/109
Smoking(yes/no)	39/198
Age (years)	27.5 ± 7.6
Age of illness onset (years)	25.0 ± 5.0
Disease duration (months)	7.3 ± 4.3
Education (years)	13.2 ± 2.9
Weight (kg)	57.8 ± 8.0
BMI (kg/m^2^)	20.6 ± 1.9
PANSS-positive	23.0 ± 1.8
PANSS-negative	20.9 ± 1.9
PANSS-general	35.0 ± 3.9
PANSS-total	79.5 ± 4.9

BMI, body mass index; PANSS, the Positive and Negative Syndrome Scale, including positive symptoms, negative symptoms, general psychopathology subscales.

The genotype and allele frequencies of 4 SNPs are shown in Table 2. No significant deviation was found between observed values and predicted values from the Hardy-Weinberg equilibrium (Table 2).

**Table 2:** **Genotype and allele frequencies of the FTO gene and the hardy Weinberg equilibrium test**

SNP	Genotype	n	%	Allele	Frequency	HWE	
						***χ*** ^2^	***P***
rs9939609	AA	4	1.7	A	0.11		
	AT	46	19.4	T	0. 89	0.354	0.552
	TT	187	78.9				
rs8050136	AA	3	1.3	A	0.10		
	AC	43	18.1	C	0.90	0.107	0.743
	CC	191	80.6				
rs1421085	CC	3	1.3	C	0.11		
	CT	45	19.0	T	0.89	0.030	0.862
	TT	189	79.7				
rs9930506	GG	10	4.2	G	0.17		
	AG	59	24.9	A	0.83	2.553	0.110
	AA	168	70.9				

HWE: The Hardy-Weinberg equilibrium test.

To calculate the extent of linkage disequilibrium (LD) in pairwise combinations of the 4 SNPs, we calculated D’ and r^2^, the normalized LD statistic for all possible pairs of SNPs. The pairwise D’ and r^2^ values are shown in Table 3. Strong LD among the two SNPs (rs9939609 and rs8050136) was observed (r^2^ > 0.33) (Table 3).

**Table 3:** **Linkage disequilibrium analysis of FTO SNPs**

D’	rs9939609	rs8050136	rs1421085	rs9930506
r^2^				
rs9939609	----	0.977	0.607	0.111
rs8050136	0.855	----	0.382	0.038
rs1421085	0.006	0.002	----	0.547
rs9930506	0.000	0.000	0.008	----

The top right is the value of D’, and the bottom left is the value of r^2^.

At baseline, body weight and BMI of TT homozygotes were lower than those of A allele carriers in rs9939609, and body weight of AA homozygotes was higher than those of G allele carriers in rs9930506 (p’s < 0.05). After 6 months of risperidone treatment, body weight and BMI of TT homozygotes were lower than those of A allele carriers in rs9939609 (p’s <0.01), body weight and BMI of CC homozygotes were lower than those of A allele carriers in rs8050136 (p’s < 0.05), and body weight of AA homozygotes was higher than those of G allele carriers in rs9930506 (p’s < 0.05).There was no significant difference in body weight and BMI between genotypes in other gene loci (p’s >0.05) (Table 4).

**Table 4:** **Comparison of body weight and BMI between different genotypes**

Characteristics	rs9939609				rs8050136				rs1421085				rs9930506			
	AA + AT	TT	T or x ^2^	P	AA + AC	CC	t or x ^2^	p	CC + CT	TT	t or x ^2^	p	GG + AG	AA	t or x ^2^	p
Male/female (n)	27/23	101/86	0.000	0.999	25/21	103/88	0.003	0.959	25/23	103/86	0.090	0.764	35/34	93/75	0.423	0.516
Age (years)	29.0 ± 7.5	26.9 ± 6.8	−1.819	0.07	29.1 ± 7.7	26.9 ± 6.8	−1.912	0.057	27.8 ± 7.5	27.3 ± 6.9	−0.427	0.67	27.7 ± 7.3	27.2 ± 6.9	−0.487	0.627
Age of illness onset (years)	26.2 ± 5.5	24.7 ± 4.7	1.949	0.053	26.3 ± 5.6	24.7 ± 4.7	1.772	0.81	24.8 ± 5.2	25.1 ± 4.9	−0.393	0.695	24.9 ± 4.8	25.0 ± 5.0	−0.174	0.86
Disease duration (months)	6.6 ± 4.9	7.5 ± 4.2	−1.334	0.183	6.5 ± 4.2	7.6 ± 4.3	−1.517	0.131	7.1 ± 3.9	7.4 ± 4.4	−0.427	0.670	7.5 ± 4.4	7.5 ± 4.3	0.411	0.682
Education (years)	13.3 ± 2.5	13.2 ± 3.0	−0.246	0.806	13.3 ± 2.6	13.2 ± 3.0	−0.296	0.767	13.2 ± 3.0	13.2 ± 2.9	−0.104	0.917	13.6 ± 3.1	13.0 ± 2.9	−1.223	0.223
Baseline																
Weight (kg)	63.0 ± 10.8	59.5 ± 10.1	2.14	0.033*	62.6 ± 10.9	59.7 ± 10.2	1.707	0.089	61.7 ± 10.1	59.9 ± 10.4	1.065	0.288	58.2 ± 10.1	61.1 ± 10.4	−2.012	0.045*
BMI(kg/m^2^)	22.4 ± 3.2	21.2 ± 2.8	2.383	0.020*	22.3 ± 3.3	21.3 ± 2.8	1.955	0.055	22.1 ± 3.0	21.3 ± 2.9	1.637	0.103	21.0 ± 3.0	21.7 ± 2.9	−1.7272	0.085
Six months																
Weight (kg)	71.7 ± 10.3	67.0 ± 9.8	2.949	0.004*	71.1 ± 10.4	67.2 ± 9.9	2.354	0.019*	68.9 ± 9.8	67.7 ± 10.1	0.714	0.476	65.8 ± 10.0	68.9 ± 9.984	−2.132	0.034*
BMI (kg/m2)	25.5 ± 3.0	23.9 ± 2.7	3.628	0.000*	25.4 ± 3.0	24.0 ± 2.7	3.009	0.003*	24.7 ± 2.8	24.2 ± 2.8	1.215	0.226	23.8 ± 3.0	24.5 ± 2.7	−1.786	0.075

BMI, body mass index.

*P<0.05.

**Figure 1:**
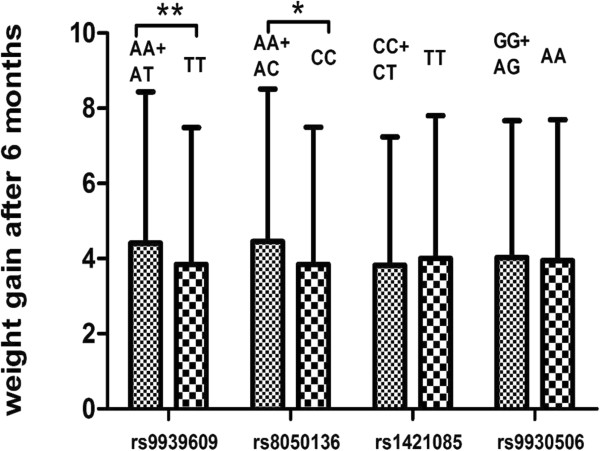
**Comparison of weight gain after 6 months of risperidone treatment between different genotypes.**

After controlling for age, gender, age of illness onset, disease duration, weight at baseline and education, weight gain of TT homozygotes was lower than those of A allele carriers in rs9939609 (p < 0.01), and weight gain of CC homozygotes was lower than those of A allele carriers in rs8050136 (p = 0.01). There was no significant difference in weight gain at 6 months between genotypes in other gene loci (p’s >0.05) (Figure 1). Further, stepwise multiple regression model was developed to identify relevant gene predictors of weight gain at 6 months when other important potential confounding variables, including age, gender, education, disease duration, age of illness onset, weight at baseline were taken into consideration. Only rs9939609 entered into the regression model (R2 change = 0.039, df, 1,229, F change = 10.886, p = 0.001) (Table 5).

**Table 5:** **Multiple regression analysis: rs9939609 predicts weight gain at 6 months after controlling for age, gender, education, disease duration, age of illness onset and weight at baseline**

Predictors	R2 change	df	F change	P
Step 1: age, gender, education, disease duration, age of illness onset, weight at baseline	0.131	6,230	5.782	<0.001
Step 2: rs9939609	0.039	1,229	10.886	0.001

Potential confounding variables including age, gender, education, disease duration, age of illness onset and weight at baseline were entered first (forced entry, Step 1); rs9939609, rs8050136, rs1421085 and rs9930506 genes were considered next (stepwise, Step 2), and rs9939609 gene was the only variable that entered into the regression model.

## Discussion

To our knowledge, this is the first study to examine the relationship between the four FTO SNPs and antipsychotic-associated weight gain in drug naïve, Chinese Han patients with first episode schizophrenia. Our study suggested that SNPs rs9939609 and rs8050136 of FTO gene might play an important role in weight gain after 6 months of risperidone treatment. Our study did not find significant relationships between SNPs rs9930506 and rs1421085 of FTO gene and antipsychotic-associated weight gain.

Our results suggested that A allele in rs9939609 and rs8050136 could be a risk allele of weight gain in first-episode schizophrenia; the findings are consistent with the results from a previous study in a healthy Japanese human sample [25], but they found no significant difference in BMI across different medications. Other studies also found that the FTO gene is associated with obesity or obesity-related diseases in non-schizophrenia populations [5, 6]. Liu et al. [8] identified positive associations between SNPs rs9939609 and rs8050136 of FTO gene and the risk to develop type 2 diabetes in the Chinese Han population. Ahmad et al. [26] found that the SNP rs8050136 polymorphism was associated with obesity and other metabolic problems in healthy US women. Few studies have investigated the relationship between FTO SNP polymorphisms and antipsychotic-associated weight gain with inconsistent findings [27–30]. For example, Tiwari et al. [31] found that the SNP rs9922047 polymorphism of FTO gene was associated with percent weight gain in patients with schizophrenia; Perez-Iglesias et al. [28] found the magnitude of weight gain was similar among 3 genotypes of the SNP rs9939609 of FTO gene in patients with schizophrenia after 1 year of antipsychotic treatment.

The FTO gene is the first and most robustly replicated gene that is related to body weight and fat mass [32, 33]. However, the underlying mechanisms remain to be unclear. FTO gene is highly expressed in the brain, particularly in the hypothalamus, which governs energy balance by regulating appetite and food intake [28]. Furthermore, it has been described that the FTO gene encodes a 2-oxoglutarate-dependent nucleic acid demethylase [34, 35], which is a transcription coactivator related to energy homeostasis [34]. The rs9939609 is in the first intron of FTO gene. Frayling et al. [6] first reported that rs9939609 is association with obesity-related traits in both adults and children of European descents. Chang et al. [16] found that rs9939609 A allele is strongly associated with obesity and BMI in the Chinese population; another study in Chinese and Malays who lived in Singapore also reported a significant association between rs9939609 and obesity [17]. For the first time, our study found that s9939609 is associated with weight gain after risperidone treatment in patients with schizophrenia.

Our research has some limitations. First, the sample size is relatively small and larger studies are needed to confirm our findings. Second, patients on other antipsychotics were not included in the study. Third, there was lack of a healthy control group. Therefore it is unclear if the effect sizes of the genetic markers (4 SNPs) in patients with schizophrenia are the same as in the general population. Lastly, potential confounding variables that could contribute to weight gain such as dosage of risperidone; environmental factors like physical activity, diet, employment status, as well as other genetic risk factors were not measured in the present study. In order to better understand the effect of genetic factors on antipsychotic-associated weight gain, future studies should examine gene-environmental interaction and gene-gene interaction to explore the role of FTO gene polymorphisms in antipsychotic-associated weight gain.

## Conclusion

Our findings suggest that FTO SNP rs9939609 seem to be related to weight gain after risperidone treatment in Chinese Han patients with first episode schizophrenia.

## Abbreviations

FTO gene: At-mass and obesity-associated gene
SNP: Single nucleotide polymorphisms
PCR-RFLP: Polymerase chain reaction-restriction fragment length polymorphism
BMI: Body mass index
AAPs: Atypical antipsychotics
GWAS: Genome Wide Association Studies
PANSS: Positive and Negative Syndrome Scale
HWE: Hardy-Weinberg equilibrium
LD: Linkage disequilibrium
DSM-IV: Diagnostic and statistical manual of mental disorders, fourth edition.
